# Efficacy of a curcumin extract (Curcugen™) on gastrointestinal symptoms and intestinal microbiota in adults with self-reported digestive complaints: a randomised, double-blind, placebo-controlled study

**DOI:** 10.1186/s12906-021-03220-6

**Published:** 2021-01-21

**Authors:** Adrian L. Lopresti, Stephen J. Smith, Alethea Rea, Shavon Michel

**Affiliations:** 1 Clinical Research Australia, Perth, Western Australia 6023 Australia; 2 College of Science, Health, Engineering and Education, Murdoch University, Perth, Western Australia 6150 Australia; 3 DolCas Biotech, LLC, Landing, NJ 07850 USA

**Keywords:** Curcumin, Turmeric, Gastrointestinal symptoms, IBS, Microbiota

## Abstract

**Background:**

There is preliminary evidence to suggest curcumin can alleviate digestive symptoms in adults with self-reported digestive complaints and irritable bowel syndrome. However, in all these trials, curcumin was used as a component of a multi-herbal combination and there were consistent concerns associated with risk of bias in most studies. The goal of this study was to investigate the effects of a curcumin extract (Curcugen™) on gastrointestinal symptoms, mood, and overall quality of life in adults presenting with self-reported digestive complaints. Moreover, to determine the potential therapeutic mechanisms of action associated with curcumin, its effects on intestinal microbiota and small intestinal bowel overgrowth (SIBO) were examined.

**Methods:**

In this 8-week, parallel-group, double-blind, randomised controlled trial, 79 adults with self-reported digestive complaints were recruited and randomised to receive either a placebo or 500 mg of the curcumin extract, Curcugen™. Outcome measures included the Gastrointestinal Symptom Rating Scale (GSRS), intestinal microbial profile (16S rRNA), Depression, Anxiety, and Stress Scale – 21 (DASS-21), Short Form-36 (SF-36), and SIBO breath test.

**Results:**

Based on self-report data collected from 77 participants, curcumin was associated with a significantly greater reduction in the GSRS total score compared to the placebo. There was also a greater reduction in the DASS-21 anxiety score. No other significant between-group changes in self-report data were identified. An examination of changes in the intestinal microbial profile and SIBO test revealed curcumin had no significant effect on these parameters. Curcumin was well-tolerated with no significant adverse events.

**Conclusions:**

The curcumin extract, Curcugen™, administered for 8 weeks at a dose of 500 mg once daily was associated with greater improvements in digestive complaints and anxiety levels in adults with self-reported digestive complaints. Compared to the placebo, there were no significant changes in intestinal microbiota or SIBO; however, further research using larger samples and testing methods that allow more detailed microbial analyses will be important. An investigation into other potential mechanisms associated with curcumin’s gastrointestinal-relieving effects will also be important such as examining its influence on the intestinal barrier function, inflammation, neurotransmitter activity, and visceral sensitivity.

**Trial registration:**

Australian New Zealand Clinical Trials Registry, Trial ID. ACTRN12619001236189. Registered 6 September 2019.

**Supplementary Information:**

The online version contains supplementary material available at 10.1186/s12906-021-03220-6.

## Background

Turmeric, the dried or fresh rhizome of the plant *Curcuma longa L.*, has been extensively used in traditional medicine. In Ayurvedic medicine (a system of traditional medicine originating from India), turmeric is believed to have many medicinal properties including strengthening the overall energy of the body, relieving gas, dispelling worms, improving digestion, regulating menstruation, dissolving gallstones, and relieving arthritis. In many South Asian countries, it is also used as an antiseptic for cuts, burns, and bruises; and as an antibacterial agent [1].

Turmeric contains several phytochemicals such as turmerones and various polysaccharides, however, most of its therapeutic activities are believed to result from a group of yellow pigments known as curcuminoids. Curcuminoids are a mixture of curcumin, demethoxycurcumin, bisdemethoxycurcumin, and cyclocurcumin. Of these, curcumin is the principal curcuminoid and has attracted the greatest interest in the scientific literature [2]. Curcumin has been shown to target multiple signalling molecules with most of its benefits believed to be due to its antioxidant and anti-inflammatory effects [3]. There is evidence to suggest it may be beneficial for the treatment of pain and arthritis [4, 5], metabolic syndrome [6], depression [7, 8], and cognitive impairment [9]. Interest in the effects of curcumin on digestive health is also accumulating and there is preliminary evidence to suggest that it may be beneficial. In a review by Lopresti [10], it was postulated that curcumin may be helpful for the treatment of gastrointestinal (GI) conditions because of its multiple effects on the GI system including its influence on intestinal microbiota, intestinal permeability, gut inflammation and oxidative stress; and bacterial, parasitic, and fungal infections. In a meta-analysis of 5 clinical trials on the use of curcumin for reducing symptoms associated with irritable bowel syndrome (IBS), it was concluded that there was overall evidence of efficacy [11]. However, in all these trials, curcumin was used as a component of a multi-herbal combination and there were consistent concerns associated with risk of bias in most studies. Beneficial effects from curcumin have also been identified in inflammatory bowel diseases and functional gastrointestinal diseases (FGID) [12–14].

The goal of this 8-week, randomised, double-blind, placebo-controlled trial was to investigate the effects of a curcumin extract (Curcugen™) on GI symptoms, mood, and overall quality of life in adults presenting with self-reported digestive complaints. In addition, to determine the potential therapeutic mechanisms of action associated with curcumin, its effects on intestinal microbiota and small intestinal bowel overgrowth (SIBO) were examined. In several animal trials [15–17] and one small human trial [18], curcumin ingestion was associated with changes in gut microbiota. For example, in an animal trial, curcumin intake for 6 weeks resulted in increases in the Firmicutes/ Bacteroidetes ratio compared to a vehicle control [15], and in a human 8-week trial, turmeric and curcumin supplementation was associated with a 7 and 69% increase in detected species respectively, over time [18]. In another small trial comprising eight healthy adults, the ingestion of curry with turmeric significantly increased the area under the curve of breath hydrogen and shortened small-bowel transit time compared with a curry mixture without turmeric [19]. Despite this preliminary evidence of the GI effects of curcumin, and its potential to impact on intestinal microbiota, there has been no trial specifically examining the GI effects of the curcumin extract, Curcugen™. However, it was hypothesised that based on previous research into turmeric and other curcumin extracts, Curcugen™ may be associated with improvements in digestive symptoms and such changes could be due to its influence on intestinal microbiota.

## Methods

### Study design

**Fig. 1:**
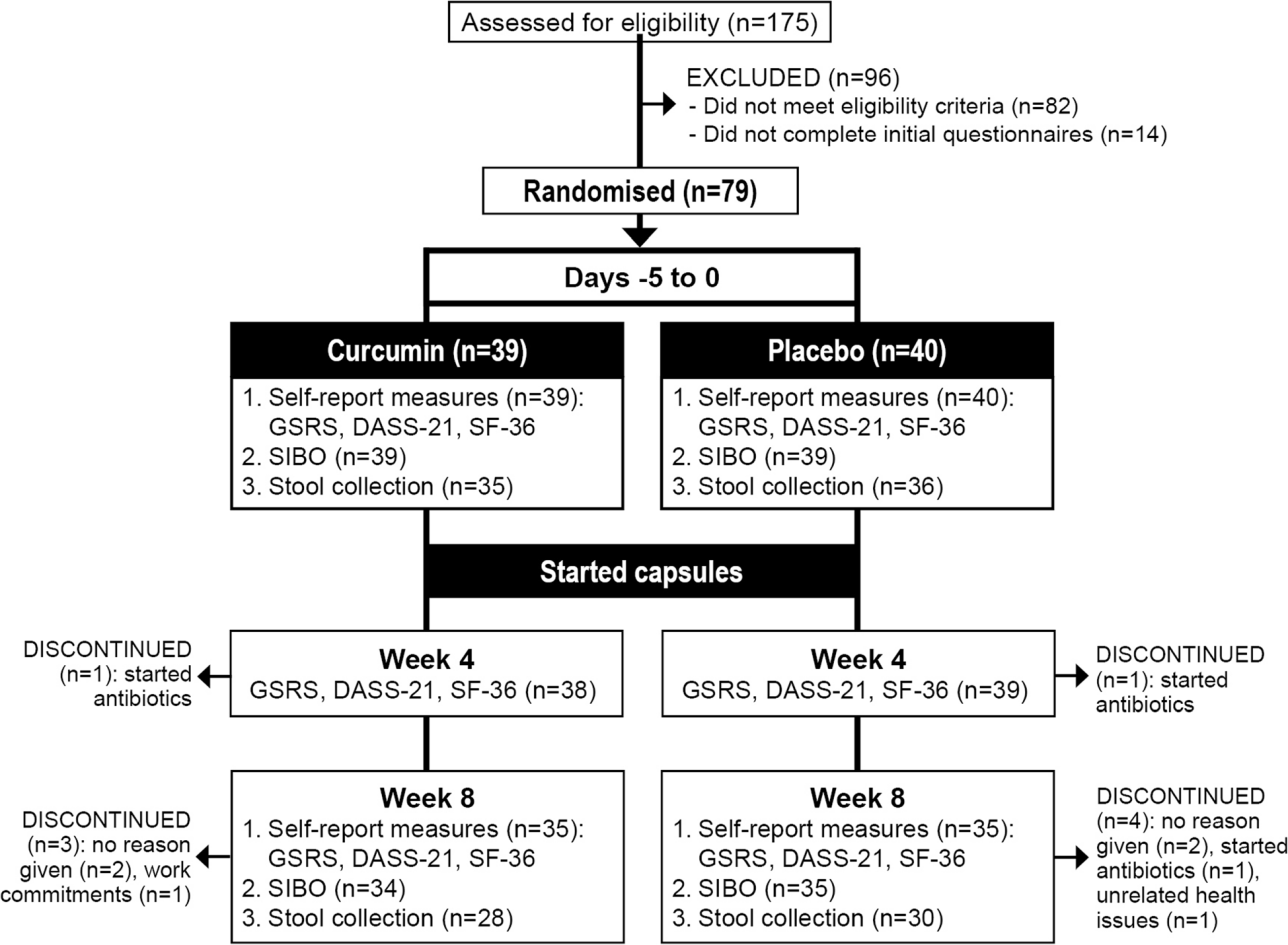
Systematic Illustration of Study Design

This was a two-arm, parallel-group, 8-week, randomised, double-blind, placebo-controlled trial (Fig. 1). The trial protocol was prospectively registered with the Australian New Zealand Clinical Trials Registry (Trial ID. ACTRN12619001236189) and approved by the Human Research Ethics Committee at the National Institute of Integrative Medicine (approval number 0056E_2019). To estimate the required sample size (based on a single outcome variable), an a priori power analysis was undertaken. Based on a previously conducted placebo-controlled study on adults with digestive disturbances using a herbal combination containing turmeric [20], an effect size of 0.6 was predicted. Assuming a type one error rate (alpha) of 5% and a power of 80%, the number of participants required per group to find an effect for the total score on Gastrointestinal Symptom Rating Scale (GSRS) was estimated as 36. After allowing for a 10% drop out rate, we aimed to recruit at least 40 participants per group.

### Recruitment and randomisation

Participants were recruited across Australia through social media advertisements between September and December 2019. Interested participants were directed to a website page that provided information about the study and a request to complete an online screening questionnaire. This questionnaire assessed for current digestive symptoms, history of medical and psychiatric disorders, medication use, alcohol, nicotine and other drug use, herb and vitamin intake, and pregnancy/breastfeeding status. If judged as likely eligible, volunteers participated in a phone interview with an investigator. The phone interview comprised a series of questions to further obtain information relating to the eligibility criteria and to acquire further demographic details. Suitable participants then completed online versions of the Gastrointestinal Symptom Rating Scale (GSRS), Depression, Anxiety, and Stress Scale (DASS-21), Short-Form-36 (SF-36), and an informed consent form. Using a randomisation calculator (http://www.randomization.com), eligible and consenting participants were randomly assigned to one of two groups (curcumin or placebo). The randomisation structure comprised 10 randomly-permuted blocks, containing 8 participants per block. Participant identification numbers were allocated according to the order of participant enrolment in the study. All capsules were packed in identical bottles labelled by two intervention codes (held by the study sponsor until final data analysis). Participants and study investigators were blind to treatment group allocation until all outcome data were collected and analysed. No financial compensation was provided to participants for participating in this study, although after the study, participants allocated to the placebo condition were given a complimentary 8-week supply of curcumin capsules.

### Participants

#### Inclusion criteria

Male and female participants aged 18 to 65 years, with self-reported symptoms of gastrointestinal complaints were recruited for this study. Participants had a GSRS average rating of between 2 (mild-severity gastrointestinal discomfort) and 5 (moderately-severe gastrointestinal discomfort). All participants were medication-free for at least 4 weeks except for the use of the contraceptive pill and no more than once a week use of pain-relieving medications. Volunteers had had no plan to change their diet or commence new treatments over the study period and had a body mass index (BMI) between 20 and 35. Participants were also fluent in English and have consented (via an online consent form) to all pertinent aspects of the trial.

#### Exclusion criteria

Participants were ineligible to participate in the study if they were diagnosed with a medical condition including, but not limited to: diabetes, hyper/hypotension, cardiovascular disease, gallbladder disease/gallstones, biliary disease, endocrine disease, psychiatric disorder, or neurological disease (Parkinson’s, Alzheimer’s disease, intracranial haemorrhage, head or brain injury). Participants diagnosed by a medical practitioner with a GI disease (e.g., inflammatory bowel diseases and FGID) were also unable to participate in this study. Participants who reported consuming greater than 14 standard alcoholic drinks per week, a current or 12-month history of illicit drug abuse, used antibiotics in the 4-weeks before study commencement, were taking curcumin supplements, or were currently taking supplements that may affect digestive function (unless they were on a stable dose for 60 days before the baseline assessment) were also ineligible for the study. Women who were pregnant, breastfeeding, or intending to fall pregnant were also ineligible to participate in the study.

### Interventions

Placebo and curcumin capsules were matched for colour, shape, and size. The active treatment, supplied by DolCas Biotech, LLC., contained 500 mg of a standardised curcuminoids extract (Curcugen™). Curcugen™ is a dispersible, 98.5% turmeric-based ingredient, containing 50% curcuminoids, 1.5% essential oils and other native turmeric molecules. By increasing the surface area of the curcuminoids, polar-resins facilitate the self-dispersion of otherwise lipophilic curcuminoids for greater access to absorptive surfaces and extended plasma retention.

The placebo capsules contained the same excipients as the active tablet (microcrystalline cellulose). All capsules were manufactured and packed in a Good Manufacturing Practice facility. All participants were instructed to take one capsule daily with 250 ml of water immediately before sleep for 8 weeks. Medication adherence was monitored by asking participants to count remaining capsules at weeks 4 and 8. The effectiveness of participant treatment blinding was assessed by asking participants to predict group allocation (placebo, curcumin, or uncertain) at the completion of the study. Curcumin and placebo capsules were mailed to participants with directions for use provided on capsule bottles. An information sheet was also provided to participants with details about how to take the capsules. This information was also verbally communicated to participants during their screening telephone interview.

### Outcome measures

#### Primary outcome measure

##### Gastrointestinal symptom rating scale (GSRS) total score

The GSRS is a self-report, 15-item questionnaire that measures the severity of a wide range of gastrointestinal symptoms. Questions are rated on a 7-point scale ranging from no discomfort at all (1) to very severe discomfort (7). A total score is calculated by averaging the ratings provided on all questions. The GSRS has good psychometric properties and in several clinical trials has shown to be sensitive to treatment effects [21, 22]. The GSRS was completed at baseline, week 4, and week 8.

##### Microbial profile

At baseline and week 8, participants collected a stool sample at home after a morning bowel motion. Participants were instructed to collect approximately 1 g of stool into a collection tube (Zymo DNA/RNA Shield faecal collection tube) and return samples via express post to our office for later analysis. DNA/RNA Shield reagent is a DNA and RNA stabilisation solution for nucleic acids that preserves the genetic integrity and expression profiles of samples at ambient temperatures and completely inactivates infectious agents. The DNA and RNA stabilisation solution also prevents degradation from freeze-thaw cycling and unexpected freezer failures. Samples were stored in a − 20-degree Celsius freezer until later analysis. Intestinal microbiota via 16S rRNA (V3-V4 region) gene sequencing was undertaken by Australian Genome Research Facility. Paired-ends reads were assembled by aligning the forward and reverse reads using PEAR (version 0.9.5). Primers were identified and trimmed. Trimmed sequences were processed using Quantitative Insights into Microbial Ecology (QIIME 1.8.4) USEARCH (version 8.0.1623) and UPARSE software. Using USEARCH tools, sequences were quality filtered, full length duplicate sequences were removed and, were sorted by abundance. Singletons or unique reads in the data set were discarded. Sequences were clustered followed by chimera filtered using “rdp_gold” database as a reference. To obtain the number of reads in each operational taxonomic unit (OU) U, reads were mapped back to OTUs with a minimum identity of 97%. Taxonomy was assigned using QIIME. OTU changes in intestinal bacteria at the phylum and family level and changes in microbial diversity (Shannon and Simpson diversity index) from baseline to week 8 were examined.

#### Secondary outcome measures

##### Gastrointestinal symptom rating scale (GSRS) cluster scores

In addition to a GSRS total score, five symptom clusters can also be calculated comprising scores for reflux, abdominal pain, indigestion, diarrhoea, and constipation.

##### Depression, anxiety, and stress scale – 21 (DASS-21)

The DASS-21 is a validated self-report measure assessing symptoms of stress, anxiety, and depression [23]. Twenty one questions are rated on a 4-point scale (0–3), ranging from never to almost always (lower scores indicate a reduction in symptoms). Sub-scale scores for depression, anxiety, and stress are calculated. The DASS-21 was completed at baseline, week 4, and week 8.

##### Short Form-36 health survey (SF-36)

The SF-36 is a self-report measure assessing quality of life. Scores are calculated for eight areas including (1) energy/fatigue, (2) physical functioning, (3) bodily pain, (4) general health perceptions, (5) physical role functioning, (6) emotional role functioning, (7) social role functioning, and (8) emotional wellbeing. The SF-36 is a commonly-used outcome measure of quality of life with strong psychometric properties [24, 25]. Scoring for the SF-36 was based on the algorithm developed by RAND Health Care [26]. The SF-36 was completed at baseline, week 4, and week 8.

##### Small intestinal bacterial overgrowth (SIBO)

A 3-h SIBO home breath test was conducted at baseline and week 8. After a 24-h preparatory diet, a lactulose substrate was ingested, and breath samples were collected every 20 min for 3 h. Tests using the QuinTron BreathTracker were used to measure levels of carbon dioxide (CO_2_), hydrogen, and methane in a single sample of breath (alveolar air). CO_2_ levels below 1.5% were considered invalid breath collections. Positive, borderline, and negative SIBO results were calculated based on the following criteria:

- Elevated hydrogen results: Increases of hydrogen greater than 20 ppm (ppm) over the lowest preceding value within the first 100 min were classified as positive results, and increases occurring 100–120 min later were classified as borderline.
- Elevated methane results: Increases of methane greater than 12 ppm over the lowest preceding value within the first 100 min were classified as positive results, and increases occurring 100–120 min later were classified as borderline.
- Elevated combined results: Increases of combined hydrogen and methane gas values greater than 15 ppm over the lowest preceding value within the first 100 min were classified as positive results, and increases occurring 100–120 min later were classified as borderline.

##### Adverse events

Safety and tolerability of supplement intake by participants were examined at weeks 4 and 8 through an online question querying adverse effects that were believed to be associated with supplement intake. Participants were also requested to contact researchers immediately if any adverse effects were experienced.

### Statistical analysis

An independent samples T-test was used to compare demographic variables across the two treatment groups for continuous variables, and Pearson’s Chi-square was used to compare categorical data. To evaluate self-report outcome measures, a repeated-measures analysis of variance (ANOVA) was used to compare within-group changes over time (weeks 0, 4, and 8) and group (curcumin versus placebo) x time interaction effects. A Cohen’s d was also calculated to examine effect sizes. The Shapiro-Wilk normality test was conducted to examine the normality of group data. This demonstrated that data were not normally distributed, and this was not corrected by data transformations. However, a repeated-measures ANOVA was considered the most appropriate option for statistical analyses as it is relatively robust to violations of normality [27]. Where necessary, degrees of freedom were adjusted using the Greenhouse-Geisser approach to correct for violations of the sphericity assumption. Data from participants were included in analyses of self-report outcomes if questionnaire data were obtained at week 4 [last observation carried forward from week 4 for missing values].

To examine SIBO results, a Mann-Whitney U-Test was conducted to examine categorical differences (elevated, borderline, and negative) for hydrogen, methane, and combined gases between the two groups (curcumin and placebo) at the two time points (baseline and week 8). A Wilcoxon signed ranks test was also conducted to evaluate within-group changes in SIBO results over time (baseline and week 8) for the two groups (curcumin and placebo).

Changes in intestinal bacteria were analysed using a permutational multivariate analysis of variance (PERMANOVA) to evaluate the difference between pre- and post-measures, by group (placebo vs curcumin), at the phylum level. A simple t-test on the Simpson and Shannon diversity index to compare the change in bacterial diversity by group at the phylum to genus levels was also conducted. Moreover, a principal component analysis (PCA) was completed to explore the relationship between samples.

A further exploratory analysis was conducted to examine changes in selected individual bacteria over time. Based on the results of several systematic reviews examining differences in gut microbiota between adults with irritable bowel syndrome and healthy adults, the following gut microbiota was chosen for further exploratory analysis: Bacteroidetes (Phyla), Firmicutes (Phyla), Clostridia (class), Enterobacteriaceae (family), Bacteroides (genus), Clostridiales (genus), Faecalibacterium (genus), and Bifidobacterium (genus) [28–30]. Moreover, an exploratory analysis was conducted to examine the relationship between change in diversity index and change in GSRS total score. Based on available data from both groups, a Pearson’s correlation coefficient was calculated at the phylum to genus levels. All data were analysed using SPSS (version 26; IBM, Armonk, NY) and R (version 3.6.2 with analysis of bacterial diversity using the package vegan).

## Results

### Study population

#### Baseline questionnaire and demographic information

From 175 people who completed the initial online screening questionnaire, 79 volunteers met the eligibility criteria and agreed to participate in the study. Self-report data from 77 participants who completed at least week-4 questionnaires were used for statistical analyses of self-report outcome measures. Two participants, one from each group, failed to consume the minimum number of required tablets (i.e., consumed less than 80% of tablets). However, data from these participants were included in the statistical analyses as removal of their results did not significantly influence statistical outcomes. SIBO results were collected from 78 participants at baseline and 69 participants at week 8. A basal hydrogen value of more than 16 ppm is suggestive of non-adherence to the preparatory diet [31]. This occurred in 1 participant at baseline and 9 participants at week 8. However, all collected data were analysed as removal of scores from these participants did not significantly affect statistical results. Stool samples were collected by 71 participants at baseline and 58 participants at week 8. However, valid pre and post stool samples were only available for 50 participants (25 from each group). This was because only one stool sample (either pre or post) was collected/returned by the participant, or an inadequate sample was collected. Statistical analyses of between-group changes were conducted on stool results where both pre and post collections were conducted.

Baseline data for participants are detailed in Table 1. There were no statistically-significant, between-group differences in baseline outcome measures except for the SF-36 pain score, where participants in the curcumin group reported a lower pain score (indicating a higher pain disability). A total of 9 participants withdrew from the study and there were no significant differences in dropout rates between the groups. Reasons for withdrawal included no reason given (*n* = 4), the commencement of antibiotics (*n* = 3), excess work commitments (*n* = 1), and unrelated health issues (*n* = 1). No participants withdrew from the study due to reported adverse events associated with capsule intake.

**Table 1:** Baseline demographic and outcome measures of participants

	Placebo	Curcumin	*p*-value
	*n* = 40	*n* = 39	
Age			
Mean	43.20	40.69	.325 ^a^
SE	1.74	1.84	
BMI			
Mean	24.80	25.53	.404 ^a^
SE	0.62	0.61	
Gender (n)			
Male	6 (15%)	4 (10%)	.869^b^
Female	34 (85%)	35 (90%)	
Marital Status (n)			
Single	10 (25%)	15 (91%)	.198 ^b^
Married	30 (75%)	24 (38%)	
Educational Level (n)			
Secondary	16 (27%)	22 (56%)	.137 ^b^
Tertiary	15 (63%)	14 (36%)	
Post-graduate	9 (10%)	8 (12%)	
Exercise Level (n)			
Never/Rarely	7 (18%)	5 (13%)	.947 ^b^
1 to 2 times a week	8 (20%)	8 (21%)	
3 to 5 times a week	15 (37%)	38 (8%)	
6+ times a week	10 (25%)	28 (18%)	
Duration of gastrointestinal problems (n)			
< 6 months	2 (5%)	1 (3%)	.148 ^a^
6 to 12 months	1 (10%)	0 (0%)	
1–2 years	2 (2%)	4 (10%)	
2 to 5 years	3 (8%)	9 (23%)	
5 to 10 years	14 (35%)	6 (15%)	
10+ years	18 (45%)	18 (46%)	
GSRS: Total score			
Mean	2.63	2.89	.106 ^a^
SE	0.10	0.12	
GSRS: Abdominal pain			
Mean	2.50	2.5	.998 ^a^
SE	0.14	0.13	
GSRS: Reflux			
Mean	2.06	2.14	.764 ^a^
SE	0.18	0.19	
GSRS: Diarrhoea			
Mean	2.71	2.65	.872 ^a^
SE	0.24	0.24	
GSRS: Indigestion			
Mean	3.15	3.52	.118 ^a^
SE	0.15	0.18	
GSRS: Constipation			
Mean	2.44	3.06	.059 ^a^
SE	0.20	0.26	
DASS-21: Total score			
Mean	22.35	23.23	.830 ^a^
SE	2.7	3.08	
DASS-21: Depression			
Mean	6.15	5.74	.798 ^a^
SE	1.02	1.21	
DASS-21: Anxiety			
Mean	5.00	5.64	.598 ^a^
SE	0.83	0.88	
DASS-21: Stress			
Mean	11.2	11.85	.730 ^a^
SE	1.22	1.41	
SF-36: Physical functioning			
Mean	88.38	88.67	.605 ^a^
SE	2.27	2.39	
SF-36: Role limitations due to physical health			
Mean	70.63	70.51	.990 ^a^
SE	6.26	6.55	
SF-36: Role limitations due to emotional problems			
Mean	60.8	69.28	.306 ^a^
SE	5.97	5.67	
SF-36: Energy/fatigue			
Mean	42.38	43.85	.748 ^a^
SE	3.5	2.92	
SF-36: Emotional wellbeing			
Mean	68.4	73.23	.169 ^a^
SE	2.38	2.54	
SF-36: Social functioning			
Mean	75.5	73.9	.750 ^a^
SE	3.83	3.2	
SF-36: General health			
Mean	59.25	65.26	.206 ^a^
SE	3.77	2.79	
SF-36: Pain			
Mean	74.55	63.36	.017 ^a^
SE	2.95	3.47	
SIBO	*n* = 39	*n* = 39	
Elevated hydrogen (n)			
Negative	12 (31%)	13 (39%)	.115 ^b^
Borderline	7 (18%)	4 (12%)	
Positive	20 (51%)	16 (48%)	
Elevated methane (n)			
Negative	16 (41%)	18 (46%)	.446 ^b^
Borderline	5 (13%)	8 (21%)	
Positive	18 (46%)	13 (33%)	
Elevated combined (n)			
Negative	3 (8%)	8 (21%)	.256 ^b^
Borderline	8 (21%)	6 (15%)	
Positive	28 (72%)	25 (64%)	

a = Independent samples t-test; b = Chi-square Test

### Outcome measures

#### GSRS total score (primary outcome measure)

**Fig. 2:**
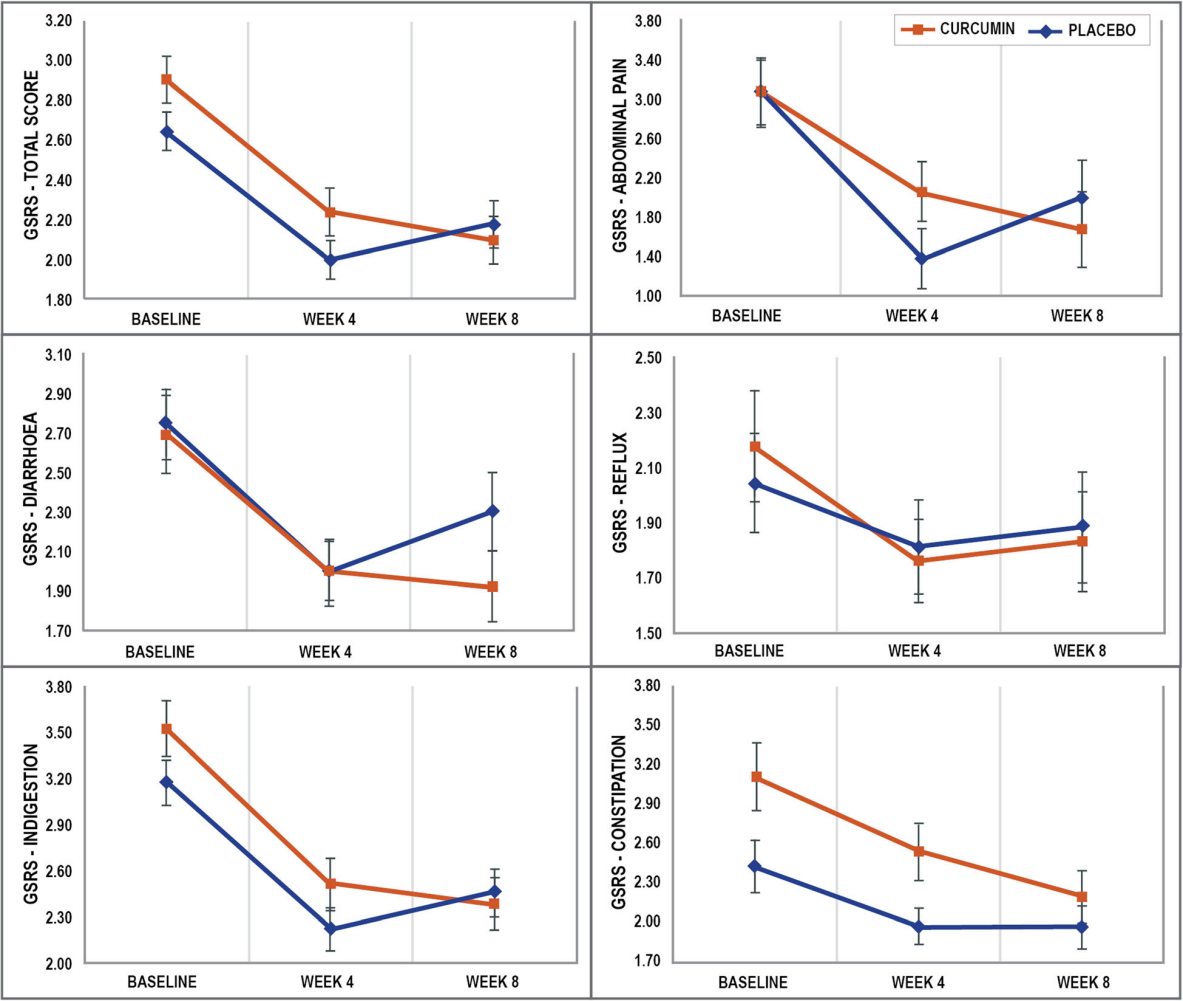
Change in GSRS Scores (error bars depict standard error)

Changes in GSRS total score across the two treatment groups and repeated measures ANOVA significance levels are detailed in Table 2 and Fig. 2. Reductions in the GSRS total score from baseline to week 8, indicating an improvement in symptoms, were significantly greater in the curcumin group than the placebo group (F_2,150_ = 3.96, *p* = .021, ES = .57). Curcumin (F_2,74_ = 36.74, *p* < .001) and placebo (F_2,76_ = 25.06, *p* < .001) administration was associated with statistically-significant reductions in the GSRS total score over time. Within-group analyses revealed that the majority of the changes in the GSRS total score occurred in the first 4 weeks of treatment as there were statistically-significant reductions in the GSRS total score from baseline to week 4 for both the curcumin (F_1,37_ = 37.08, *p* < .001) and placebo (F_1,38_ = 45.39, *p* < .001) groups. However, between-group contrasts revealed a significant group x time interaction from week 4 to week 8 for GSRS total score (F_1, 75_ = 6.433, *p* = .013). From weeks 4 to 8, there was a non-significant decrease in GSRS total score in the curcumin group (F_1,37_ = 2.92, *p* = .096) and a non-significant increase in GSRS total score in the placebo group (F_1,38_ = 3.56, *p* = .067).

**Table 2:** Changes in GSRS scores over time

			Baseline	Week 4	Week 8	Repeated Measures ANOVA		Cohen’s D effect size
						*p*-value time effects	*p*-value time x group interaction	
GSRS: Total score	Placebo (*n* = 39)	Mean	2.65	2.00	2.18	<.001	.021	.57
		SE	0.10	0.10	0.12			
	Curcumin (*n* = 38)	Mean	2.91	2.24	2.10	<.001		
		SE	0.12	0.12	0.12			
GSRS: Abdominal pain	Placebo (*n* = 39)	Mean	2.52	1.85	2.10	<.001	.124	.14
		SE	0.14	0.12	0.15			
	Curcumin (*n* = 38)	Mean	2.52	2.12	1.97	.001		
		SE	0.13	0.12	0.15			
GSRS: Reflux	Placebo (*n* = 39)	Mean	2.04	1.81	1.88	.350	.638	.20
		SE	0.18	0.17	0.20			
	Curcumin (*n* = 38)	Mean	2.17	1.76	1.83	.021		
		SE	0.20	0.15	0.18			
GSRS: Diarrhoea	Placebo (*n* = 39)	Mean	2.75	2.00	2.31	<.001	.314	.24
		SE	0.24	0.16	0.21			
	Curcumin (*n* = 38)	Mean	2.70	2.01	1.93	<.001		
		SE	0.24	0.18	0.18			
GSRS: Indigestion	Placebo (*n* = 39)	Mean	3.18	2.22	2.46	<.001	.114	.41
		SE	0.15	0.14	0.16			
	Curcumin (*n* = 38)	Mean	3.53	2.52	2.39	<.001		
		SE	0.18	0.17	0.17			
GSRS: Constipation	Placebo (*n* = 39)	Mean	2.43	1.98	1.97	.009	.160	.40
		SE	0.20	0.14	0.16			
	Curcumin (*n* = 38)	Mean	3.10	2.54	2.20	<.001		
		SE	0.26	0.22	0.20

#### Microbial profile (primary outcome measure 2)

**Fig. 3:**
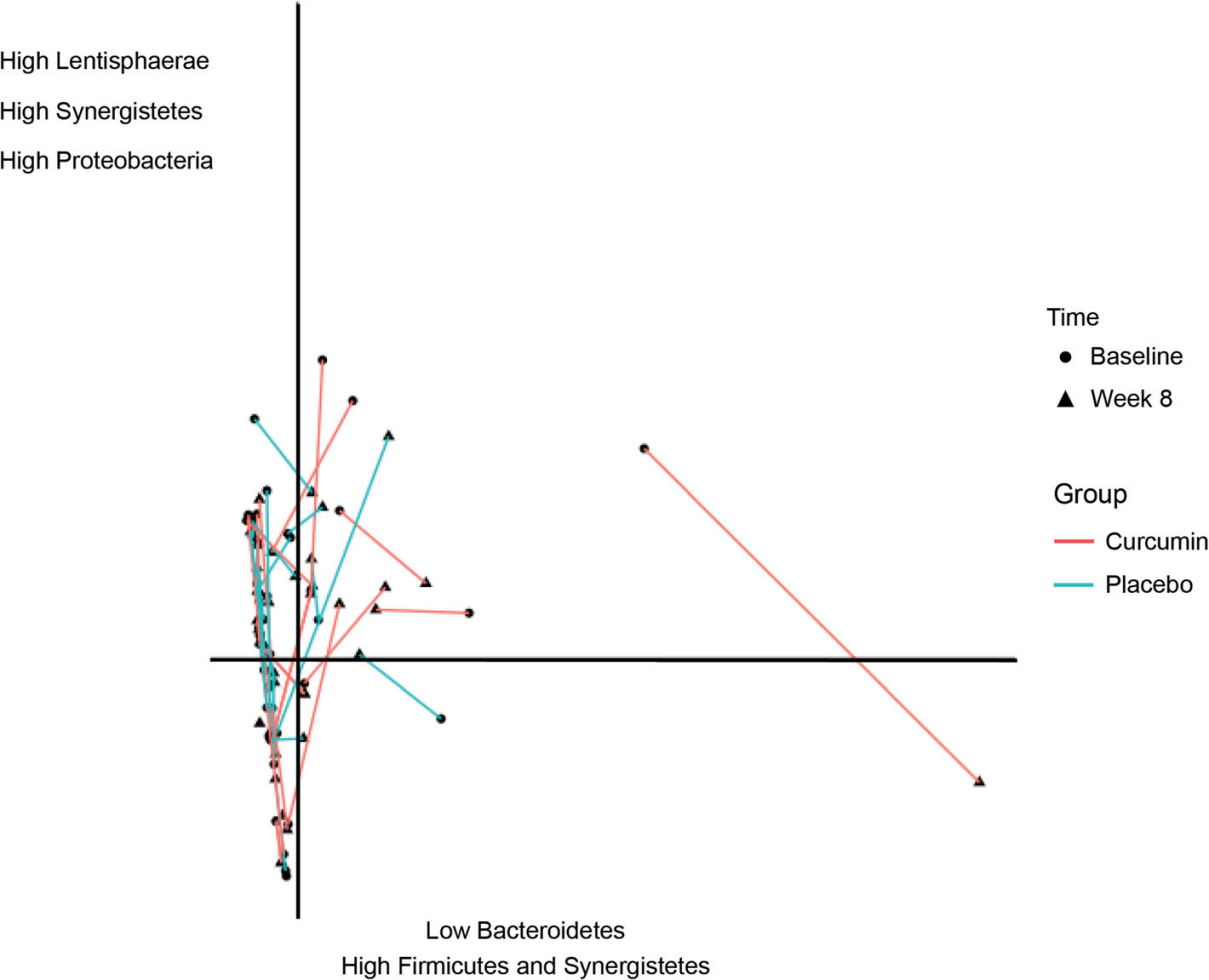
Principal Components Analysis (PCA) of Changes in Bacterial Concentrations (Phyla) for the Curcumin and Placebo Groups Over Time (Time A = baseline; Time B = week 8)

Based on the results of the PERMANOVA, there was a non-significant between-group interaction in changes in intestinal bacteria at the phylum level between the placebo and curcumin groups (F_1,48_ = 1.730, *p* = .169). The visual inspection of the PCA (Fig. 3) also revealed that there was no systematic movement in phyla concentrations in the baseline and week 8 samples.

The results of independent samples t-tests examining changes in microbial diversity (Shannon and Simpson index) over time between the two groups revealed there were no statistically-significant between-group differences in changes in the Shannon and Simpson index at the phylum, class, order, or family level (see supplementary Table 1). However, at the genus level, there was a statistically-significant difference between the curcumin and placebo groups for the Shannon (*p* = .020) and Simpson index (*p* = 0.06). This was characterised by a decrease in the Shannon [T(24) = − 2.20, *p* = .038] diversity index in the curcumin group. However, as there were baseline differences in diversity between the two groups, a univariate analysis of variance was conducted with baseline diversity scores included as a covariate. This revealed that between-group differences in changes in diversity scores were no longer significantly different for the Shannon (F_2,47_ = 3.69, *p* = .061) and Simpson diversity (F_2,47_ = 1.92, *p* = .171) index.

T-tests were conducted to examine OTU changes in selected individual bacteria from the phylum to species level. As detailed in supplementary Table 1 there were no statistically-significant, between-group differences in changes in the selected intestinal bacteria over time.

An exploratory analysis was conducted to examine the relationship between change in the Shannon and Simpson diversity index and change in GSRS total score (supplementary Table 2). Based on the Pearson’s correlation coefficient, there was no significant relationship between change in diversity index at any microbial level and GI symptomatic improvements as measured by the GSRS total score.

#### GSRS subscale scores (secondary outcome measure 1)

Changes in GSRS cluster scores for the two treatment groups and repeated measures ANOVA significance levels are detailed in Table 2. Concerning changes in all GSRS cluster scores (abdominal pain, reflux, diarrhoea, indigestion, and constipation) across the 8-week intervention, there were no statistically-significant, between-group differences. However, in the curcumin group there were statistically-significant reductions in all GSRS cluster scores comprising abdominal pain (F_2,74_ = 7.15, *p* = .001), reflux (F_2,74_ = 4.04, *p* = .021), diarrhoea (F_2,74_ = 8.90, *p* < .001), indigestion (F_2,74_ = 28.88, *p* < .001), and constipation (F_2,74_ = 14.69, *p* < .001). In the placebo group, there were statistically-significant reductions in GSRS cluster scores for abdominal pain (F_2,76_ = 13.59, *p* < .001), diarrhoea (F_2,76_ = 8.42, *p* < .001), indigestion (F_2,76_ = 23.08, *p* < .001), and constipation (F_2,76_ = 5.06, *p* = .009), but not reflux (F_2,76_ = 1.06, *p* = .350).

#### DASS-21 (secondary outcome measure 2)

Changes in the DASS-21 scores for the two treatment groups and repeated measures ANOVA significance levels are detailed in Table 3. Reductions in the DASS-21 total (F_2,150_ = 3.19, *p* = .044, ES = .46) and anxiety scores (F_2,150_ = 3.85, *p* = .023, ES = .53) were significantly greater in the curcumin group than the placebo group. However, there were no between-group differences for changes in the DASS-21 depression (F_2,150_ = 1.02, *p* = .363, ES = .26) or stress (F_2,150_ = 1.87, *p* = .157, ES = .35) scores. In the curcumin group, there were statistically-significant reductions in all DASS-21 scores; total (F_2,74_ = 14.25, *p* < .001), depression (F_2,74_ = 5.58, *p* = .006), anxiety (F_2,74_ = 10.81, *p* < .001), and stress (F_2,74_ = 9.93, *p* < .001) scores compared to baseline levels. In the placebo group, there was a statistically-significant reduction in the DASS-21 stress score (F_2,76_ = 5.29, *p* = .007), but not the total (F_2,76_ = 2.96, *p* = .058), anxiety (F_2,76_ = .995, *p* = .375), or depression (F_2,76_ = .869, *p* = .423) scores compared to baseline levels.

**Table 3:** Changes in DASS-21 and SF-36 scores over time

			Baseline	Week 4	Week 8	Repeated Measures ANOVA		Cohen’s D effect size
						*p*-value time effects	*p*-value time x group interaction	
DASS-21: Total Score	Placebo (*n* = 39)	Mean	22.87	19.71	19.28	.058	.044	.46
		SE	2.71	2.69	3.03			
	Curcumin (*n* = 38)	Mean	23.42	13.95	13.89	<.001		
		SE	3.16	1.81	1.86			
DASS-21: Depression	Placebo (*n* = 39)	Mean	6.31	5.33	5.44	.423	.363	.26
		SE	1.03	1.00	1.15			
	Curcumin (*n* = 38)	Mean	5.79	3.42	3.47	.006		
		SE	1.25	0.72	0.64			
DASS-21: Anxiety	Placebo (*n* = 39)	Mean	5.08	4.67	4.26	.375	.023	.53
		SE	0.85	0.86	0.83			
	Curcumin (*n* = 38)	Mean	5.68	3.05	2.74	<.001		
		SE	0.90	0.57	0.59			
DASS-21: Stress	Placebo (*n* = 39)	Mean	11.49	9.18	9.59	.007	.157	.35
		SE	1.22	1.16	1.30			
	Curcumin (*n* = 38)	Mean	11.95	7.47	7.68	<.001		
		SE	1.45	0.82	0.95			
SF-36: Physical functioning	Placebo (*n* = 39)	Mean	88.08	89.74	91.92	.170	.250	.11
		SE	2.31	2.77	2.60			
	Curcumin (*n* = 38)	Mean	86.58	90.92	89.08	.019		
		SE	2.45	1.70	2.32			
SF-36: Role limitations due to physical health	Placebo (*n* = 39)	Mean	69.87	80.13	84.87	.137	.814	.03
		SE	6.37	5.60	4.77			
	Curcumin (*n* = 38)	Mean	69.74	84.87	81.41	.008		
		SE	6.68	2.32	5.09			
SF-36: Role limitations due to emotional problems	Placebo (*n* = 39)	Mean	59.79	66.67	77.79	.019	.476	.14
		SE	6.03	6.13	5.53			
	Curcumin (*n* = 38)	Mean	70.24	81.61	82.45	.060		
		SE	5.74	4.82	5.00			
SF-36: Energy/fatigue	Placebo (*n* = 39)	Mean	41.79	46.92	52.05	.003	.073	.11
		SE	3.55	3.26	3.59			
	Curcumin (*n* = 38)	Mean	44.47	56.58	52.63	<.001		
		SE	2.93	2.80	3.48			
SF-36: Emotional wellbeing	Placebo (*n* = 39)	Mean	67.90	71.69	79.47	.016	.272	.20
		SE	2.39	2.93	2.06			
	Curcumin (*n* = 38)	Mean	73.47	79.47	74.26	.047		
		SE	2.59	2.06	2.58			
SF-36: Social functioning	Placebo (*n* = 39)	Mean	74.87	85.13	89.92	<.001	.983	.01
		SE	3.88	3.00	2.81			
	Curcumin (*n* = 38)	Mean	74.18	85.05	88.97	<.001		
		SE	3.27	3.50	2.60			
SF-36: General health	Placebo (*n* = 39)	Mean	58.72	63.59	65.26	.002	.919	.08
		SE	3.83	3.33	3.38			
	Curcumin (*n* = 38)	Mean	65.13	69.47	70.53	.031		
		SE	2.86	2.77	3.13			
SF-36 - Pain	Placebo (*n* = 39)	Mean	74.41	79.15	81.51	.018	.023	.43
		SE	3.06	2.82	2.54			
	Curcumin (*n* = 38)	Mean	62.66	77.61	77.29	<.001		
		SE	3.49	2.78	2.99			

#### SF-36 subscale scores (secondary outcome measure 3)

Changes in SF-36 scores for the two treatment groups and repeated measures ANOVA significance levels are detailed in Table 3. Improvements in the SF-36 pain score (F_2,150_ = 3.85, *p* = .023, ES = .43) were greater in the curcumin group than the placebo group. However, as pain scores were significantly different at baseline between the two groups, a univariate analysis of variance with baseline pain scores included as a covariate was conducted. This revealed that between-group differences in changes in pain scores were no longer significantly different (F_2,74_ = 0.175, *p* = .677). For all other SF-36 subscale scores, there were no statistically-significant, between-group differences.

#### SIBO (secondary outcome measure 4)

SIBO tests results are detailed in Table 4. A basal hydrogen value of more than 16 ppm is suggestive of non-adherence to the preparatory diet [31]. This occurred in 1 participant at baseline and 9 participants at week 8. However, all collected data were included in analyses as removal of data from these participants did not significantly affect statistical outcomes. Based on the Mann-Whitney U-Test, there were no statistically-significant, between-group differences in the percentage of participants scoring in the elevated, borderline, or negative categories for either hydrogen, methane, or combined gases at either baseline or week 8. A Wilcoxon signed ranks test was conducted to examine changes in SIBO results from baseline to week 8. There were no statistically-significant changes in SIBO from baseline to week 8 in the placebo group for either hydrogen (z = −.502, *p* = .616), methane (z = −.427, *p* = .670) or combined gases (z = −.884, *p* = .377); or the curcumin group for hydrogen (z = − 1.539, *p* = .124), methane (z = − 1.734, *p* = .083) or combined gases (z = −.262, *p* = .794).

**Table 4:** Changes in SIBO results over time

	Baseline				Week 8				Placebo	Curcumin
	Placebo (*n* = 39)	Curcumin (*n* = 39)	Difference	*p-value	Placebo (*n* = 34)	Curcumin (*n* = 35)	Difference	*p-value	^#^*p*-value	^#^*p*-value
Hydrogen										
Elevated	51%	36%	15%	.069	50%	46%	4%	.447	.616	.124
Borderline	18%	10%	8%		21%	11%	9%			
Negative	31%	54%	−23%		29%	43%	−13%			
Methane										
Elevated	46%	33%	13%	.395	44%	54%	−10%	.247	.670	.083
Borderline	13%	21%	−8%		15%	14%	0%			
Negative	41%	46%	−5%		41%	31%	10%			
Combined										
Elevated	72%	64%	8%	.323	79%	74%	5%	.420	.377	.794
Borderline	21%	15%	5%		15%	3%	12%			
Negative	8%	21%	−13%		6%	23%	−17%			

* Mann-Whitney U-Test (between-group differences as time points); # Wilcoxon signed ranks test (within-group changes in SIBO results from baseline to week 8)

#### Intake of supplements

At week 8, participants recorded their quantity of remaining capsules. Two participants reported taking less than 80% of their capsules over the 8 weeks, although the consistency of use over the 8 weeks could not be ascertained.

#### Adverse events

No significant adverse events were reported by participants and no participant withdrew from the study due to concerns associated with supplement intake. The frequency of adverse effects is detailed in supplementary Table 3. These were no significant differences in reported adverse effects between the two groups [placebo (11) and curcumin (6)] and reported adverse effects were of mild severity.

#### Efficacy of participant blinding

To evaluate the efficacy of condition concealment over the study, participants were asked at the completion of the study to predict condition allocation (i.e. placebo, curcumin, or uncertain). Efficacy of group concealment was high as only 31% in the curcumin group and 29% in the placebo group correctly guessed treatment allocation.

## Discussion

In this 8-week, randomised, double-blind, placebo-controlled trial, the administration of a curcumin extract (Curcugen™) at a dose of 500 mg a day was associated with a greater improvement in overall GI symptoms in adults presenting with self-reported digestive complaints compared to the placebo. After 8 weeks of treatment, there was an average 28% reduction in overall digestive symptoms in the curcumin group compared to 18% in the placebo group. Moreover, from weeks 4 to 8, Curcugen™ was associated with improvements in GI symptoms, while the placebo group experienced a worsening in symptoms. In relation to symptomatic improvements, curcumin was also associated with significant reductions in abdominal pain, reflux, diarrhoea, indigestion, and constipation; however, improvements were not significantly different to those observed in the placebo group. Improvements in quality of life and mood were also observed after curcumin administration; however, except for a greater reduction in anxiety, changes were not significantly different from the placebo group. In adults supplemented with curcumin, there was a 52% reduction in anxiety symptoms compared to a 16% reduction in the placebo group. These results suggest curcumin administration was associated with improvements in overall GI symptoms and reductions in anxiety. Curcumin was well-tolerated with no significant differences in adverse effects between the curcumin and placebo groups.

To examine the potential mechanisms associated with curcumin’s influence on the GI system, changes in intestinal microbiota and SIBO were examined over the 8-week intervention. Compared to the placebo, there were no significant changes in microbial diversity (from the phylum to genus level) or in examined selected microbiota comprising Bacteroidetes (Phyla), Firmicutes (Phyla), Clostridia (class), Enterobacteriaceae (family), Bacteroides (genus), Clostridiales (genus), Faecalibacterium (genus), and Bifidobacterium (genus). These intestinal bacteria were chosen for examination as differences in these bacteria have been regularly observed in adults with IBS [28–30]. An examination of the effects of curcumin on SIBO also revealed curcumin did not influence SIBO rates. These results suggest that although curcumin was associated with improvements in GI symptoms (and anxiety levels) in adults with self-reported digestive complaints, changes in intestinal bacteria were not responsible for its therapeutic mechanisms of action. Importantly, despite increasing interest in the relationship between microbial diversity and GI conditions, in this study, increases in microbial diversity (from the phylum to genus level) were not associated with GI symptomatic improvements. This finding provides further confirmation that in this examined population of adults with self-reported digestive complaints, therapeutic actions of curcumin were not via its impact on intestinal bacteria. However, it is important to note that 16S rRNA analysis was utilised in this study which lacks the resolution to identify microbial changes at lower taxonomic classifications (e.g., species and strain levels). Other potential mechanisms associated with curcumin’s GI-supporting effects include its influence on intestinal barrier function, inflammation, neurotransmitter activity, and visceral sensitivity; mechanisms which have been identified as compromised in adults with GI disturbances. In a review by Ghosh et al. [32], it was demonstrated that curcumin decreases the translocation of gut bacteria-derived lipopolysaccharide (LPS) from the intestinal lumen into circulation by maintaining the integrity of the intestinal barrier. In particular, curcumin may modulate intestinal barrier function at several layers including the lumen (via its effect on intestinal alkaline phosphatase), the mucus layer, the epithelium (through its influence on the expression of tight junction proteins), and antibacterial peptides (via its expression of anti-microbial peptides). In an acute study, curcumin supplementation reduced gastrointestinal damage and associated pro-inflammatory cytokine activity in adults exposed to exercise and heat stress [33]. Moreover, curcumin has anti-inflammatory effects and this presents as another mechanism associated with its therapeutic effects. Increased concentrations of systemic LPS are identified in several intestinal and extra-intestinal diseases [34–37]. Curcumin has been identified in animal studies to attenuate LPS-induced immune response and tissue damage [38–40]. Disturbances in the activity of the hypothalamus-pituitary-adrenal (HPA) axis have also been identified in people with IBS and FGID [41–43]. Curcumin has been shown in animal studies to influence HPA activity, and in human trials, is associated with lower concentrations of cortisol [44, 45]. Disturbances in serotonergic activity have also been implicated in GI diseases and altered serotonergic signalling may lead to both intestinal and extraintestinal symptoms in IBS [46]. This presents as another mechanism of action associated with curcumin as it has been shown to influence serotonergic activity [47, 48].

### Limitations and directions for future research

Even though there were improvements in GI symptoms in adults with self-reported GI complaints, these results should be cautiously generalised to adults presenting with diagnosed GI disorders until confirmation in future clinical trials. How the severity of digestive complaints in the population recruited in this study compares to healthy individuals without GI complaints, or people with diagnosed GI disorders, could not be evaluated as there are no published norms for the GSRS. However, in a study on 516 adults with gastroesophageal reflux disease, an average GSRS total score of 2.27 was identified [21]. This suggests that the population of recruited participants had a mild-to-moderate severity of GI symptoms, as their baseline GSRS score was 2.78 (a higher scores indicates increasing severity of symptoms). Further examination of changes in GI symptoms utilising other outcome measures will be helpful in future trials to provide further analysis of symptomatic changes. Examples include the Bristol Stool Scale [49], clinician-administered questionnaires, measures of colonic transit time and stool frequency, and provocation tests for the evaluation of visceral sensitivity [50, 51].

In this study, curcumin was administered as a once-daily dose delivering 500 mg of a patented curcumin extract (Curcugen™). This equates to 250 mg of curcuminoids daily. Research confirms that there are significant differences in the bioavailability and composition of curcumin extracts. Curcugen™ is derived from a turmeric oleoresin base with a 50% curcuminoid concentration. In an unpublished study, it was associated with a 39-fold increase in free curcumin compared to standard curcumin. Given the differences in the profile and bioavailability of curcumin extracts, the GI effects of curcumin should be cautiously extended to other curcumin extracts until further confirmatory research is conducted.

In future trials, the efficacy of curcumin should be examined using different doses, frequency of dosing, and treatment duration. This will help to identify optimal treatment regimens for people with GI disorders. Even though no significant changes in intestinal microbiota were identified in this study, different dosages and treatment periods may lead to microbial changes. Moreover, microbial changes may occur in adults with clearly defined and diagnosed GI disorders including IBS, other FGIDs, and inflammatory bowel disease. In this study, adults with self-reported GI complaints with no previous diagnosis of a GI disorder were recruited. In addition, more detailed analysis of microbial changes at different taxonomic levels (e.g., species or strain level) may have revealed changes over time. However, for this greater resolution to occur accurately, whole-genome testing is preferred [52]. Other factors that may have affected the lack of change in intestinal microbiota over time included the recruitment of people taking the contraceptive pill, the inclusion of volunteers with a BMI in the overweight and obese range, and limitations associated with our ability to monitor or control for dietary changes over time. The recruitment of larger samples sizes may also be required to elucidate microbial changes associated with curcumin use. Despite recruiting 79 volunteers, pre- and post-treatment changes in intestinal microbiota were only undertaken on 50 participants but data on GSRS changes were available for 77 participants. This has the potential to confound the results. As already discussed, despite Curcugen™ supplementation being associated with improvements in GI symptoms and reductions in anxiety, its therapeutic mechanisms of action could not be established. It will be important in future trials to examine other potential mechanisms of action of curcumin including examining its effects on intestinal barrier function, inflammation, HPA-axis activity, and serotonergic activity.

The results of this randomised, double-blind, placebo-controlled study demonstrated that the 8-week administration of a curcumin extract, Curcugen™ (500 mg daily), was associated with significant improvements in GI symptoms in adults presenting with self-reported digestive complaints. Compared to the placebo, there were also greater reductions in anxiety. Curcumin was well-tolerated with no reported significant adverse effects. An examination of the potential mechanisms associated with curcumin supplementation suggests its therapeutic effects may not be due to its influence on intestinal microbiota. However, before definitive conclusions about the effects of curcumin on intestinal bacteria can be made, further research using larger sample sizes and testing methods that allow more detailed analysis of microbial changes at lower taxonomic levels (e.g., whole-genome testing) will be important. Further studies on adults with clearly-defined GI disorders, using objective and subjective outcome measures, varying dosages and treatment durations, and differing curcumin extracts are required to elucidate the effects of curcumin on GI symptoms. Further trials are also required to examine the potential therapeutic actions of curcumin.

## Supplementary Information

**Additional file 1: Supplementary Table 1.** Changes in OTU of individual bacteria over time. **Supplementary Table 2.** Pearson’s correlation coefficient between change in diversity index and change in GSRS total score. **Supplementary Table 3.** Self-reported frequency of adverse effects.

## Data Availability

The datasets used and/or analysed during the current study are available from the corresponding author on reasonable request.

## Abbreviations

ANOVA: Analysis of variance
BMI: Body mass index
DASS-21: Depression, Anxiety, and Stress Scale – 21
FGID: Functional gastrointestinal diseases
GI: Gastrointestinal
GSRS: Gastrointestinal Symptom Rating Scale
HPA: Hypothalamus-pituitary-adrenal
IBS: Irritable bowel syndrome
LPS: Lipopolysaccharide
OUT: Pperational taxonomic unit
PERMANOVA: Permutational multivariate analysis of variance
PCA: Principal component analysis
QIIME: Quantitative Insights into Microbial Ecology
SIBO: Small intestinal bowel overgrowth
SF-36: Short Form-36
