# Supplementary material for: Efficacy of a curcumin extract (Curcugen™) on gastrointestinal symptoms and intestinal microbiota in adults with self-reported digestive complaints: a randomised, double-blind, placebo-controlled study
Source: BMC Complement Med Ther. 2021 Jan 21;21:40. doi: 10.1186/s12906-021-03220-6 (PMC7818735; doi:10.1186/s12906-021-03220-6)
Supplement: Supplementary file 1 — Additional file 1: Supplementary Table 1. Changes in OTU of individual bacteria over time. Supplementary Table 2. Pearson’s correlation coefficient between change in diversity index and change in GSRS total score. Supplementary Table 3. Self-reported frequency of adverse effects. [file 12906_2021_3220_MOESM1_ESM.docx]

##### Supplementary Table 1. Changes in OTU of individual bacteria over time

|  | | | | | | T-test | | |
| --- | --- | --- | --- | --- | --- | --- | --- | --- |
| Gut Bacteria |  | N | Mean change | Std. Error Mean | p-value, within-group | | p-value, between-group |  |
| Shannon index (Phylum) | Placebo | 25 | -0.0060 | 0.0277 | .831 | | .258 |  |
|  | Curcumin | 25 | 0.0421 | 0.0316 | .195 | |  |  |
| Simpson index (Phylum) | Placebo | 25 | -0.0154 | 0.021 | .468 | | .167 |  |
|  | Curcumin | 25 | 0.0275 | 0.0223 | .009 | |  |  |
| Shannon index (Class) | Placebo | 25 | -0.0140 | 0.0308 | .653 | | .243 |  |
|  | Curcumin | 25 | 0.0395 | 0.0332 | .246 | |  |  |
| Simpson index (Class) | Placebo | 25 | -0.0184 | 0.0212 | .394 | | .154 |  |
|  | Curcumin | 25 | 0.0260 | 0.0221 | .251 | |  |  |
| Shannon index (Order) | Placebo | 25 | -0.0133 | 0.0308 | .669 | | .267 |  |
|  | Curcumin | 25 | 0.0380 | 0.0338 | .272 | |  |  |
| Simpson index (Order) | Placebo | 25 | -0.0184 | 0.0212 | .394 | | .155 |  |
|  | Curcumin | 25 | 0.0259 | 0.0221 | .253 | |  |  |
| Shannon index (Family) | Placebo | 25 | 0.0373 | 0.0380 | .336 | | .060 |  |
|  | Curcumin | 25 | -0.0648 | 0.0370 | .092 | |  |  |
| Simpson index (Family) | Placebo | 25 | 0.0084 | 0.0144 | .566 | | .219 |  |
|  | Curcumin | 25 | -0.0139 | 0.0106 | .203 | |  |  |
| Shannon index (Genus) | Placebo | 25 | 0.0585 | 0.0499 | .252 | | .020 |  |
|  | Curcumin | 25 | -0.1185 | 0.0539 | .038 | |  |  |
| Simpson index (Genus) | Placebo | 25 | 0.0124 | 0.0137 | .375 | | .060 |  |
|  | Curcumin | 25 | -0.0221 | 0.0116 | .058 | |  |  |
| Firmicutes (Phyla) | Placebo | 25 | 0.0290 | 0.0258 | .271 | | .076 |  |
|  | Curcumin | 25 | -0.0404 | 0.0283 | .167 | |  |  |
| Bacteroidetes (Phyla) | Placebo | 25 | -0.0345 | 0.0328 | .303 | | .341 |  |
|  | Curcumin | 25 | 0.0071 | 0.0284 | .804 | |  |  |
| Clostridia (class) | Placebo | 25 | 0.0313 | 0.0259 | .238 | | .070 |  |
|  | Curcumin | 25 | -0.0395 | 0.0282 | .174 | |  |  |
| Enterobacteriaceae (family) | Placebo | 25 | -0.0019 | 0.0010 | .062 | | .231 |  |
|  | Curcumin | 25 | -0.0041 | 0.0015 | .011 | |  |  |
| Bacteroides (genus) | Placebo | 25 | -0.0393 | 0.0292 | .191 | | .341 |  |
|  | Curcumin | 25 | -0.0026 | 0.0245 | .915 | |  |  |
| Clostridiales (genus) | Placebo | 25 | -0.0017 | 0.0035 | .641 | | .243 |  |
|  | Curcumin | 25 | -0.0079 | 0.0039 | .056 | |  |  |
| Faecalibacterium (genus) | Placebo | 25 | 0.0109 | 0.0159 | .499 | | .444 |  |
|  | Curcumin | 25 | -0.0065 | 0.0159 | .689 | |  |  |
| Bifidobacterium (genus) | Placebo | 25 | -0.0007 | 0.0036 | .843 | | .422 |  |
|  | Curcumin | 25 | 0.0034 | 0.0036 | .355 | |  |  |

##### Supplementary Table 2. Pearson’s correlation coefficient between change in diversity index and change in GSRS total score

|  | Correlation (n=50) | p-value |
| --- | --- | --- |
| Shannon diversity (phylum) | -0.114 | .431 |
| Shannon diversity (class) | -0.109 | .453 |
| Shannon diversity (order) | -0.106 | .462 |
| Shannon diversity (family) | 0.090 | .536 |
| Shannon diversity (genus) | 0.099 | .495 |

##### Supplementary Table 3. Self-reported frequency of adverse effects

|  | Placebo | Curcumin |
| --- | --- | --- |
| Increased stomach pain | 3 | 1 |
| Increased body odour | 1 | 0 |
| Increased flatulence | 2 | 0 |
| Increased constipation | 1 | 1 |
| Increased nausea | 0 | 1 |
| Increased diarrhoea | 1 | 1 |
| Increased reflux | 0 | 2 |
| Increased stomach bloating | 1 | 0 |
| Increased thirst | 2 | 0 |
| Total | 11 | 6 |
